# Fibroblasts as key cellular targets in acupuncture therapy: a mechanistic perspective

**DOI:** 10.3389/fbioe.2025.1662525

**Published:** 2025-10-15

**Authors:** Shi-Wei Tu, Jun Kawanokuchi, Ken Takagi, Yang-Yang Liu, Yan-Wei Li, Jun-Yi Li, Jia-Yi Tu, Ning Ma, Yi Guo

**Affiliations:** ^1^ Department of Traditional Chinese Medicine, Qinghai University Medical College, Xining, China; ^2^ Research Center of Experimental Acupuncture Science, Tianjin University of Traditional Chinese Medicine, Tianjin, China; ^3^ Chongqing Three Gorges Medical College, Chongqing, China; ^4^ Institute of Traditional Chinese Medicine, Suzuka University of Medical Science, Suzuka, Japan; ^5^ Key Laboratory of Acupuncture-Moxibustion and Tuina Intelligent Equipment of Chongqing Administration of Traditional Chinese Medicine, Chongqing, China; ^6^ National Clinical Research Center for Chinese Medicine Acupuncture and Moxibustion, Tianjin, China; ^7^ Department of Medicine, The University of Chicago, Chicago, IL, United States

**Keywords:** acupuncture, fibroblast, mechanotransduction, mechanical force, fascia, acupoint

## Abstract

Acupuncture, a central modality in traditional Chinese medicine, is widely recognized for its clinical efficacy. In recent years, mechanistic studies have shifted from a neurocentric to a broader perspective encompassing the local microenvironment and systemic integration. This review focuses on the pivotal role of fibroblasts in mediating the mechanotransduction processes triggered by acupuncture. We comprehensively summarize current research findings on the contributions of fibroblasts to the therapeutic outcomes of acupuncture and elucidate the multidimensional mechanisms underlying them, especially through mechanical sensing, cytoskeletal remodeling, and the secretion of bioactive molecules. Acupuncture-induced mechanical forces are transmitted via the collagen fiber network to local fibroblasts within the acupoint region, activating mechanosensors and initiating cytoskeletal reorganization and extracellular matrix remodeling. Fibroblasts, in turn, secrete adenosine, hyaluronic acid, inflammatory modulators, and matrix components to mediate analgesic and anti-inflammatory effects directly. Moreover, fibroblasts engage in crosstalk with mast cells and macrophages through chemokine signaling and collagen-mediated mechanical interactions, forming a cellular interaction network that underpins a structural immunity response initiated by acupuncture. This study proposes a mechanobiochemical coupling framework that highlights fibroblasts as key mechanical transducers and regulatory hubs for the therapeutic mechanisms of acupuncture.

## 1 Introduction

As a vital component of traditional Chinese medicine, acupuncture has been practiced for thousands of years and is now widely applied to the prevention, treatment, and management of various diseases. It has been adopted in 196 countries and regions globally (Jing and Zhu, 2024). Modern research has shown that acupuncture can exert systemic regulatory effects by modulating the NEI network (Dou et al., 2021; Li et al., 2021). Neurophysiological theories emphasize the modulation of peripheral and central nervous system activity, including the regulation of neurotransmitters, endogenous opioids, and spinal cord signaling. Immunological perspectives highlight the ability of acupuncture to influence inflammatory mediators, cytokine release, and immune cell activity (Huang et al., 2004; Fan et al., 2024). With the rapid advancement of biomedical technologies, researchers have explored the potential mechanisms underlying the effects of acupuncture from multiple perspectives, including imaging, histology, molecular biology, and biomechanics. However, most of these studies have focused on neuroregulatory pathways, and the mechanisms by which acupuncture induces systemic effects through modulation of the local microenvironment remain inadequately elucidated. More recently, connective tissue-based mechanisms have gained increasing attention, suggesting that acupuncture may induce mechanical signals in the extracellular matrix and engage resident cells such as fibroblasts (Yunshan L et al., 2025; Langevin et al., 2001a; Langevin et al., 2013). These fibroblasts, as mechanosensitive and immunomodulatory cells, may serve as key mediators linking acupuncture-induced tissue mechanics with systemic physiological effects.

Fibroblasts are the most abundant cells in connective tissues, and they play a central role in tissue repair, mechanosensation, and intercellular communication (Plikus et al., 2021; Dong et al., 2025). Therefore, they have become a new focus for exploring the mechanism of acupuncture (Zhang et al., 2017; Ding et al., 2019; Fu et al., 2023; Xue and Zeng, 2024). Fibroblasts can synthesize collagen, secrete ECM components, and respond to mechanical signals. During tissue injury or repair, fibroblasts can become activated into myofibroblasts and contribute to wound healing and scar formation by contracting stress fibers, remodeling the cytoskeleton, and secreting inflammatory mediators (Younesi et al., 2024). Several studies have indicated that fibroblasts located in acupoint regions are sensitive to mechanical stimulation through integrins and G-protein-coupled receptors (GPCRs), and they can convert mechanical cues into biochemical signals via cytoskeletal remodeling. This influences both local and systemic physiological responses (Qu et al., 2020; Yunshan et al., 2025). Additionally, fibroblasts interact with mast cells, macrophages, and other cell types, potentially playing a key regulatory role in the initiation of the effects of acupuncture (Ding et al., 2009; Pakshir and Hinz, 2018; Xue and Zeng, 2022). Acupuncture-induced mechanical signals can activate fibroblasts, which, in turn, indirectly stimulate mast cells to degranulate and release mediators such as histamine and prostaglandins to amplify the biological signals generated by acupuncture (Xue and Zeng, 2024). The specific mechanisms by which fibroblasts contribute to the effects of acupuncture remain to be further explored. Key unresolved issues include the role of fibroblasts in initiating the effects of acupuncture and the direct evidence of fibroblast involvement in the mechanisms underlying them. As critical cells that bridge mechanical stimulation and biological responses, fibroblasts may offer new perspectives for understanding the modern mechanisms of acupuncture. Combining our study results, this study aims to systematically review the role of fibroblasts in the initiation of acupuncture therapy, signal transduction, and tissue repair; explore the evidence and potential pathways through which fibroblasts participate in acupuncture mechanisms; and provide a reference for future research on acupuncture mechanisms.

## 2 Overview of fibroblasts

**FIGURE 1:**
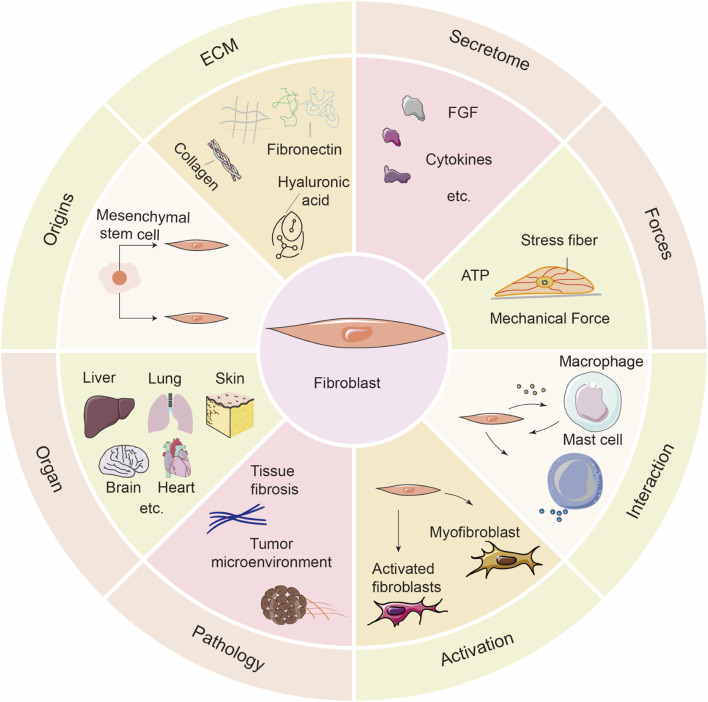
Overview of the biological characteristics and functional roles of fibroblasts. This schematic summarizes the diverse features of fibroblasts across multiple biological contexts. Origins: Fibroblasts mainly derive from mesenchymal stem cells. ECM: They are key producers of ECM components such as collagen, fibronectin, and hyaluronic acid. Secretome: Fibroblasts secrete various cytokines and growth factors, including FGFs. Forces: They respond to mechanical stimuli through stress fiber formation and ATP release. Interaction: Fibroblasts interact dynamically with immune cells such as macrophages and mast cells. Activation: Upon stimulation, fibroblasts can differentiate into activated fibroblasts and further into myofibroblasts. Pathology: Dysregulated fibroblast activation contributes to tissue fibrosis and tumor progression. Organ: Fibroblasts reside in various organs including the liver, lung, skin, brain, and heart, playing context-specific roles.

Fibroblasts are derived from mesenchymal cells during embryonic development and are the principal cellular components of connective tissues. As shown in Figure 1, fibroblasts play a key role in the synthesis and remodeling of the ECM. They produce and secrete collagen, elastin fibers, glycosaminoglycans, and reticular fibers, all of which help maintain tissue structure and function. Additionally, fibroblasts are critically involved in tissue repair following injury (Gauthier et al., 2023). Single-cell sequencing and transcriptome analysis have revealed significant differences in the gene expression profiles, functions, and distribution of fibroblasts across different organs or within the same tissue (Plikus et al., 2021). Fibroblasts are mechanosensitive cells that can sense mechanical forces and transduce these signals. Changes in the physical microenvironment and mechanical forces can alter their morphology, adhesion, migration, proliferation, and mitosis, leading to fibroblast activation. Fibroblast activation is closely associated with tissue fibrosis, immune diseases, and the tumor microenvironment (Gu et al., 2024). In addition to their mechanical sensing capabilities, fibroblasts exert immunomodulatory functions by secreting cytokines and chemokines that regulate leukocytes, mast cells, and T cells, thereby serving as key executors of structural immunity and contributing to tumor immune evasion and inflammation regulation (Wei et al., 2021). Fibroblasts also possess functions similar to those of innate immune cells. They can detect damage and pathogen-associated molecular patterns and serve as immune sentinels (Davidson et al., 2021; Shinotsuka and Denk, 2022). The multidimensional properties of fibroblasts and their role in tissue homeostasis and disease have made them a focal point in research on tissue engineering, fibrosis treatment, and mechanobiochemical coupling mechanisms.

In clinical practice, acupuncture has been demonstrated to be an effective non-pharmacological intervention for the management of pain across various conditions. Pain is a complex biological phenomenon, and its pathological process primarily involves the peripheral sensory neurons, spinal cord, and brain. However, recent studies have shown that non-neuronal cells also play a role in the pathological development of chronic pain (Shinotsuka and Denk, 2022). Fibroblasts have significant heterogeneity, and they have been identified in various regions such as the perivascular spaces of the brain and spinal cord, meninges, and choroid plexus with the advent of single-cell sequencing technology. Structurally, fibroblasts may interact directly with neurons (Dorrier et al., 2022). They can also release neurotransmitters and regulate signaling molecules involved in pain perception. One study indicated that the expressions of endogenous opioid precursor genes such as Proenkephalin, Pro-opiomelanocortin, and Prodynorphin were upregulated by 2.5–4 times when human-derived fibroblasts were exposed to a 2.5-MHz, low-intensity PRFE (150 V/m, 10 MS pulse) mechanical environment for 24 h. Additionally, the concentrations of Met-enkephalin, Leu-enkephalin, and β-endorphin in the fibroblast culture supernatant were significantly elevated. These opioid peptides can exert local analgesic and anti-inflammatory effects by acting on μ, δ, and κ receptors on nerve terminals and immune cells through a paracrine mechanism (Moffett et al., 2012). The researcher Stecco has been dedicated to studying myofascial pain and was the first to identify endogenous cannabinoid receptors CB1 and CB2 in human fascial fibroblasts (Fede et al., 2016). This finding is expected to fill some gaps in the theoretical understanding of fascial pain modulation. Activation of the CB2 receptor can inhibit the secretion of pro-inflammatory cytokines and reduce the recruitment of macrophages and neutrophils. These lead to anti-inflammatory effects. An earlier study reported that CB2 can modulate immune responses and alleviate nociceptive behaviors in both acute and chronic pain models (Elmes et al., 2004). The mechanosensitive properties of fibroblasts, their role as immune sentinels, and their potential for pain management all suggest a possible involvement in the mechanisms of action of acupuncture therapy. However, the specific pathways through which fibroblasts contribute to the effects of acupuncture still require systematic investigation.

## 3 Effect of acupuncture-induced mechanical force on fibroblasts

Traditional acupuncture involves inserting needles into the skin without the need for other exogenous stimuli. Through techniques such as lifting, thrusting, twisting, and rotating, different mechanical forces are applied to the acupoint tissue, inducing the “de qi” sensation in the patient, while the practitioner also experiences a feeling of tightness under the needle. Studies have shown that forces ranging from 240 to 280 MN can be generated during lifting and thrusting maneuvers, while twisting actions produce a tensile force in the range of 10–15 mN × mm^−1^. These tensile forces can be transmitted through the collagen fiber network to reach other areas (Zhang et al., 2008). In ancient China, medical practitioners often described the sensation of “de qi” based on their personal experiences. Classical Chinese medical literature describes the arrival of qi as follows: During acupuncture, if qi has not been obtained, the practitioner perceives no distinctive tactile sensation, akin to operating in a still and enclosed space. In contrast, when qi is obtained, the practitioner experiences a pulling or dragging sensation at the needle, comparable to the fluctuating resistance encountered when a fish bites bait. This metaphor underscores the distinctive sensory perception associated with the presence or absence of qi. These descriptions are highly subjective and can be difficult to understand for those who have not systematically studied Chinese culture. Modern researchers refer to this phenomenon as “needle grasp” and have conducted in-depth studies on it from a modern experimental perspective. Acupoints are three-dimensional structures, rich in collagen fibers, blood vessels, and nerves. What happens when an acupuncture needle is inserted into the skin? Research by the Langevin team (Langevin et al., 2001a) suggests that an acupuncture needle causes the subcutaneous collagen fibers to become disordered and entangled when inserted into the skin, resulting in the “needle grasp” phenomenon. The mechanical coupling of the needle with the connective tissue could be the biological mechanism underlying the sensation of “de qi” in acupuncture. The tension between the needle and the connective tissue transmits mechanical signals to the connective tissue cells, which regulate matrix protein synthesis through autocrine and paracrine pathways and influence cell proliferation and migration. This mechanotransduction phenomenon may partially explain the local and distant therapeutic effects of acupuncture. The team further quantified the change in the pulling force during needle removal caused by the entanglement of connective tissue (Langevin et al., 2001b). Compared with non-twisting needle manipulation, bidirectional and unidirectional twisting increased the pulling force during needle removal by 52% and 167%, respectively. The pulling force during needle removal from the acupoint was 18% higher than that from non-acupoint areas. This study further confirmed that the biological basis of the “de qi” sensation is related to the connective tissue. Continuous tracking using immunofluorescence technology revealed that approximately 30% of fibroblasts can directly connect with each other, suggesting that they may play a key role in the composition of connective tissue or in cellular communication (Langevin et al., 2004). The Langevin team focused on fibroblasts after obtaining these results and systematically studied the effects of acupuncture on these cells. They confirmed that fibroblasts in the acupoint area had increased cross-sectional area, cytoskeletal rearrangement, increased stress fibers, and the formation of lamellipodia at the cell membrane edges after 30 min of acupuncture combined with needle twisting. These changes were reversed when mechanotransduction signaling pathways, such as Rho, Rac, and purinergic signaling, were inhibited (Langevin et al., 2006; Goldman et al., 2013). This study suggests that the mechanical force of acupuncture induces cytoskeletal remodeling in fibroblasts, and the mechanism may involve the interactions of Rho and Rac with actin filaments (Langevin et al., 2006). Another study suggests that the mechanical force applied during acupuncture combined with twisting maneuvers can induce fibroblast contraction, which regulates the collagen fiber force transmission network and causes deformation of the extracellular matrix (Langevin et al., 2007). This enables the mechanical signals triggered by acupuncture to be transmitted throughout the entire connective tissue. From this, it is inferred that fibroblasts are the most important cell type among all the cells involved (Liu et al., 2020). The research team led by Chen Bo has focused on simulating the impact of acupuncture-induced mechanical forces on fibroblast secretion of chemical factors (Chen et al., 2007; 2010; 2011b; 2011a; 2012; Chen et al., 2019). After applying simulated acupuncture pressure stimulation to fibroblasts cultured *in vitro*, the secretion levels of MMP-1, TIMP1, PGE2, TNF-α, INF-β, CD30, and other factors were significantly elevated, while genes related to cell adhesion and matrix regulation, such as NOV/CCN3 and P-Selectin, were downregulated. These findings suggest that mechanical stimulation-induced biochemical changes in fibroblasts may play a crucial role in the initiation of the acupuncture effect. The mechanical signal-fibroblast-immune factor axis could be a key pathway in acupuncture therapy. As non-neuronal cells, fibroblasts are sensitive to mechanical stimuli and may contribute to the common mechanisms underlying traditional Chinese physical therapies such as acupuncture, cupping, and massage.

**FIGURE 2:**
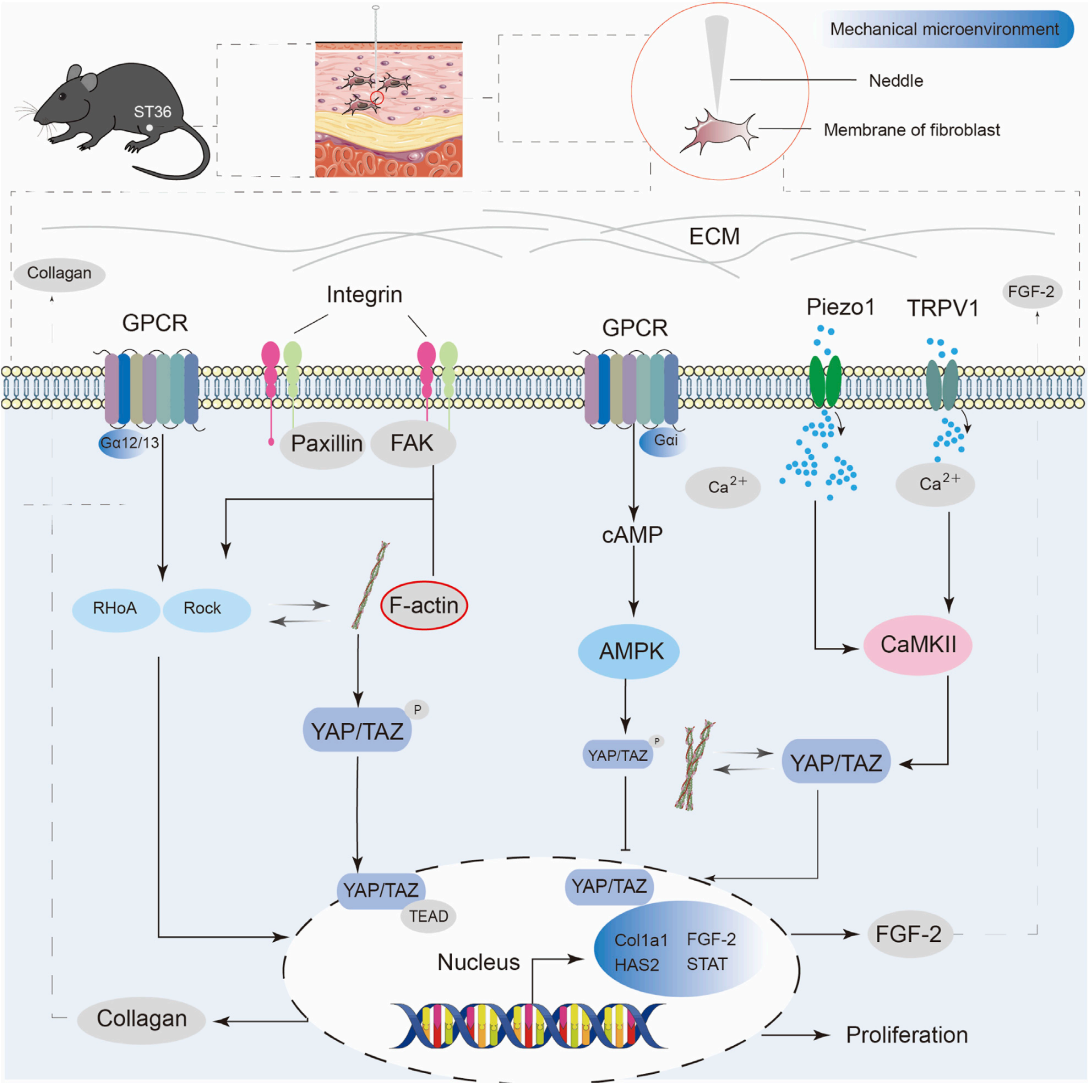
Schematic diagram of mechanotransduction pathways in fibroblasts induced by acupuncture stimulation at ST36. Mechanical stimulation from acupuncture activates multiple transmembrane receptors in fibroblasts, including GPCRs, integrins, Piezo1, and TRPV1. Integrin-FAK-paxillin signaling leads to the reorganization of F-actin, which further regulates the nuclear localization of YAP/TAZ and promotes collagen synthesis. GPCR activation can trigger the RhoA/ROCK pathway, enhancing actin cytoskeleton tension and contributing to YAP/TAZ-mediated gene transcription. Piezo1 and TRPV1 respond to mechanical force and mediate Ca^2+^ influx, which cooperates with AMPK and F-actin dynamics to regulate YAP/TAZ translocation. Nuclear YAP/TAZ activation promotes the expression of FGF-2, collagen production, and fibroblast proliferation, contributing to extracellular matrix remodeling and tissue repair following acupuncture.

From a modern biomechanical perspective, an acupuncture needle generates tensile forces that interact with the connective tissue when inserted into the body, and mechanical force is applied through manipulation. Collagen fiber entanglement transmits the mechanical signals to the cellular level. The mechanical load is sensed by integrins at the cell-matrix sites, which relay the force signals through the cytoskeletal structure to the nuclear membrane. The mechanical signal activation in fibroblasts can influence ECM synthesis and fibrosis. The activation of the DDR1-TGFβ/STAT3 and YAP/TAZ signaling axes plays a key role in this event (Chiquet et al., 2003). As shown in Figure 2, mechanosensitive ion channels on the cell membrane may also play a crucial role. For example, Piezo1 and the TRP family are sensitive to changes in membrane tension. Once these mechanosensitive ion channels are activated, they trigger Ca^2+^ influx, which in turn initiates downstream biochemical cascades. This hypothesis has been experimentally validated. The mechanical signals of acupuncture can be converted into measurable shear waves, which can be observed in both *in vivo* and *ex vivo* experiments. These shear waves stimulate various cells, including fibroblasts, to respond to mechanical stimuli. Confocal microscopy revealed Ca^2+^ oscillations, indicating that mechanical stimulation can induce cytoplasmic Ca^2+^ production in non-neuronal cells (Li et al., 2011). Some studies have used a fascial framework system composed of fibroblasts to reveal acupuncture theory, referring to it as the “fascial armor” theory. It posits that fibroblasts form the connective tissue throughout the body, acting like armor that envelops both the surface and internal organs. The ECM remodeling induced by acupuncture can affect the overall tension network and trigger systemic regulation (Plaut, 2023). The above evidence suggests that Young’s modulus (E) and shear modulus (G) of the tissue change due to the remodeling of collagen fibers and ECM during the transmission of mechanical signals. The resulting back-regulation of the distribution of force triggers the activation of downstream signaling pathways. This leads to events such as gene transcription and protein expression and couples physical with biochemical signals (Liddle and Harris, 2018; Maemichi et al., 2024; Yunshan et al., 2025).

Electroacupuncture represents one of the important advances in modern acupuncture research. It is characterized by well-defined parameters, reproducible stimulation, and reliable therapeutic efficacy, and has therefore been widely applied in many countries. Nevertheless, manual acupuncture, as the traditional and classical approach, remains particularly intriguing because it can elicit therapeutic effects solely through needle insertion and manipulation, without the aid of electrical stimulation. In this review, we focus on manual acupuncture manipulations (e.g., lifting–thrusting, twisting, and rotating), which rely purely on mechanical forces acting on connective tissue and fibroblasts. Electroacupuncture, which involves additional electrical stimulation and engages complex bioelectric and neurophysiological pathways, is beyond the scope of this article.

## 4 Analyzing the mechanisms through which fibroblasts contribute to acupuncture effects from multiple perspectives

### 4.1 Participation in tissue repair and regeneration and promoting local micro-injury healing

**FIGURE 3:**
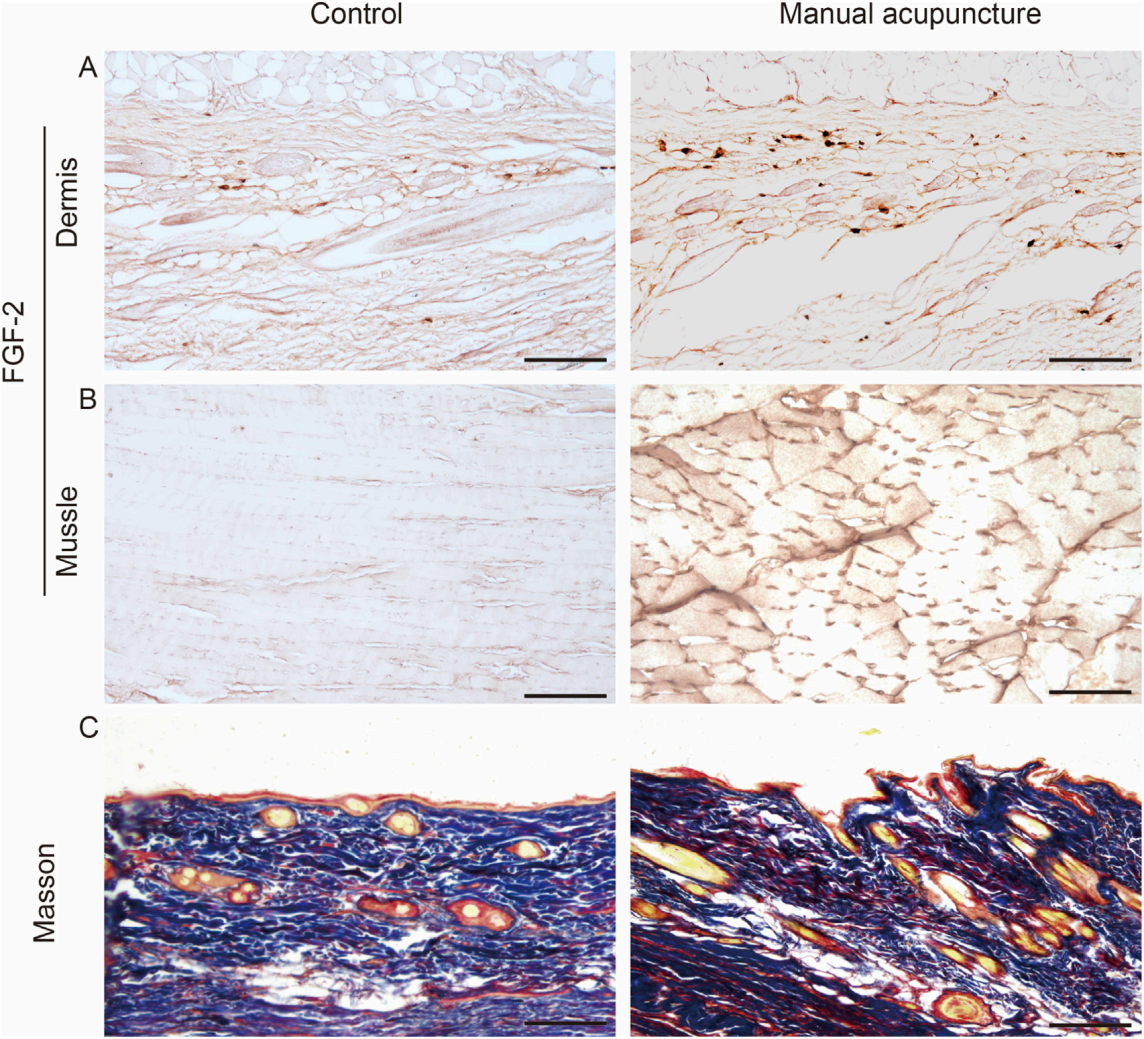
Morphological changes and FGF-2 expression in ST36 of rat after acupuncture stimulation. **(A,B)** Immunohistochemical analysis of FGF-2 expression in ST36 of rat following acupuncture. The number of FGF-2-positive cells increased in rats subjected to acupuncture stimulation. **(A)** FGF-2 expression in the fascia layer; **(B)** FGF-2 expression in the muscle layer. **(C)** Masson’s trichrome staining showing morphological changes in collagen fibers after acupuncture. Original magnification: ×200; scale bar = 100 μm.

Acupuncture is a form of aseptic inflammatory stimulation. The micro-injury induced by acupuncture causes cell membrane rupture or sub-lethal damage, leading to the release of Damage-Associated Molecular Patterns such as High Mobility Group Box 1, Heat Shock Proteins, adenosine triphosphate (ATP), and nucleic acids, among others, through active or passive mechanisms. This triggers the activation of fibroblasts, macrophages, mast cells, and other immune cells to release cytokines and chemokines that attract more white blood cells to the site (Koncz et al., 2023; Yu et al., 2023). Histopathological studies have shown that the local area of the acupuncture point goes through pathological changes such as blood vessel rupture and leukocyte infiltration after acupuncture (Zhao et al., 2020). Acupuncture increases the concentration of pro-inflammatory cytokines in normal rats. This response is also observed in inflammatory model rats, suggesting that immune factor-mediated inflammatory responses may serve as a precursor in initiating the acupuncture effect (Zhou et al., 2009; Zhang et al., 2018; 2020). Our research team has previously focused on the mechanisms underlying the initiation of acupuncture effects, with emphasis on the local acupuncture points. We have proposed the concept of the acupuncture point effect initiator, describing acupuncture points as sensors that transmit information from the body (Xu et al., 2013), amplifiers of acupuncture information transmission (Zhang et al., 2015), and transducers of mechanical-chemical coupling (Dou et al., 2022). Through techniques such as liquid phase chips, sensors, and radioimmunoassay, we have detected 55 signaling molecules in the acupuncture point that may participate in the regulation of the neuro-endocrine-immune network following acupuncture. We identified CXCL1, MCP-1, IL-1β, M-CSF, and others as key molecules potentially involved in the initiation of acupuncture effects (Ding et al., 2014; Xu et al., 2018). Studies have shown that fibroblasts can be activated through paracrine and autocrine mechanisms. Cytokines can induce fibroblast activation, prompting them to secrete factors such as CCL-2/MCP-1, CXCL8/IL-8, TGF-β1, and FGF-2. These factors, in turn, recruit circulating monocytes, macrophages, and neutrophils, promote extracellular matrix (ECM) synthesis and remodeling, and establish a self-amplifying regulatory loop (Enzerink and Vaheri, 2011). As shown in Figures 3A,B, immunohistochemical analysis revealed that acupuncture stimulation at the ST36 acupoint in rats significantly increased the expression of FGF-2 in the subfascial connective tissue and interstitial spaces of the muscle tissue. These findings suggest that fibroblasts play a key role in the initiation of acupuncture effects through immune modulation.

### 4.2 Constituting collagen fibers and transducing mechanical signals

In our previous *in vitro* study, mechanical stretching of fibroblasts induced cytoskeletal remodeling and activated mechanosensitive channels, resulting in increased expression of extracellular matrix proteins, including collagen (Tu et al., 2025). Collagen fibers are primarily composed of collagen proteins secreted by fibroblasts and are arranged in bundled or reticular patterns. As the most abundant and essential protein in the ECM, collagen plays a central role in maintaining tissue structure and function. At the microscopic level, collagen has a highly ordered triple-helical structure, which confers exceptional tensile strength and mechanical stability. Due to its anisotropic and semi-crystalline nature, collagen behaves as a liquid crystalline continuum, rendering it highly sensitive to changes in the mechanical microenvironment (Fei et al., 1998). Collagen fibers can efficiently transmit infrared light wavelengths. Their unique liquid crystalline structure supports soliton-like propagation, enabling near-lossless transmission of electromagnetic signals. Furthermore, this structure allows for the conversion of electromagnetic signals into terahertz waves, highlighting the potential role of collagen in biophysical signal transmission and energy modulation within tissues (Fei et al., 1998; Deng et al., 2005). The application of lifting-thrusting and twisting manipulation during acupuncture induces entanglement between the needle shaft and collagen fibers that results in deformation of the connective tissue (Figure 3C). The altered extracellular matrix tension enables collagen fibers to transmit signals in the form of “vibrations” or elastic waves. These mechanical signals subsequently activate fibroblasts, triggering the secretion of bioactive molecules and initiating downstream biological effects (Lyu et al., 2022). The signal transmission speed along collagen fibers is approximately three times faster than that of neural conduction (Plaut, 2023), suggesting the existence of a distinct and specialized pathway for signal propagation following acupuncture stimulation. Zeng et al. (2023) proposed that the acupuncture needle engages in a crosslinking interaction with surrounding collagen fibers upon insertion into an acupoint. However, they are unlikely to generate specific biological responses themselves due to the intrinsic properties of collagen fibers. Instead, their role in the acupuncture process may lie in transmitting mechanical signals to adjacent cells. Investigating the mechanobiological properties of cells constituting the acupoint from the perspective of cellular biomechanics may help elucidate the physicochemical coupling mechanism by which mechanical signals are transduced during acupuncture stimulation. An early study suggested that collagen fibers within acupoints may serve as the primary structural basis for sensing mechanical stimulation during acupuncture (Wang et al., 2017). Deformation of collagen fibers induced by needle insertion can activate mast cells, which in turn release bioactive substances that modulate local inflammatory responses. In another study (Ma et al., 2012), the expression of type I collagen in the acupoint area was positively correlated with skeletal muscle repair following acupuncture, further supporting the pivotal role of collagen fibers in mediating acupuncture-induced therapeutic effects. The team led by Ding Guanghong has focused on the study of mast cell-mediated acupuncture effects and found that type I collagen plays a crucial role in the therapeutic effects of acupuncture. Pre-treatment with collagenase partially antagonized the acupuncture effect, suggesting that the mechanism may be related to collagen fiber alterations that trigger mast cell degranulation (Ding and Wei, 2005; Yu, 2009; Gu et al., 2017). Mast cells collaborate with the collagen fibers and nerves at acupuncture points, and the changes in collagen fiber arrangement induced by acupuncture may play a crucial role in mast cell degranulation, leading to the release of active substances such as histamine and serotonin (5-HT). Collagen fibers can be regarded as key in the interaction between mast cells and nerve terminal receptors, contributing to the core mechanism of the analgesic effect of acupuncture (Wang et al., 2022). The regulation of mast cell activity is closely associated with endocannabinoid receptor signaling (Sugawara et al., 2012). Endocannabinoids modulate mast cell activation, maturation, degranulation, and the release of inflammatory mediators, thereby exerting anti-inflammatory and immunoregulatory effects at both local and systemic levels (Cantarella et al., 2011). These findings indicate that under physiological conditions, basal endocannabinoid signaling constrains mast cell activity and prevents excessive activation, whereas under pathological conditions such as chronic inflammation, allergic disorders, or neuropathic pain, upregulated endocannabinoid signaling inhibits the release of pro-inflammatory mediators and thereby alleviates symptoms (Angelina et al., 2020). In parallel, fibroblasts have also been shown to express components of the endocannabinoid receptor system, which may serve as a potential pathway linking fibroblast function with immune cells such as macrophages. Within the context of acupuncture research, however, the biological interactions between fibroblasts and mast cells remain insufficiently understood, and further studies are required to clarify how endocannabinoid-mediated signaling contributes to their cross-talk. A study systematically reviewed the mechanisms by which acupuncture exerts its anti-inflammatory effects at acupuncture points (Yang et al., 2023). It suggests that acupuncture may work through mechanical stimulation of the localized, fascicular, and reticular collagen fibers. After tissue deformation, mechanical signals are transmitted to connective tissue cells or activate sensory neurons or nerve fibers labeled with PROKR2 at the acupuncture point, leading to the release of chemical substances and initiating a cascade reaction that mediates acupuncture effects. Based on the above studies, fibroblasts can contribute to the formation of collagen fibers by secreting collagen proteins in addition to the secretion of chemical factors by fibroblasts or their analgesic effects. These collagen fibers construct the network structure of the acupuncture point, providing the material basis for the transmission of acupuncture mechanical signals. They are the key driving factors that promote mast cell degranulation, macrophage polarization, and fibroblast activation. Taken together, these findings suggest that fibroblasts are not merely passive structural components but active participants in acupuncture mechanisms, engaging in dynamic cross-talk with immune cells such as mast cells and macrophages. This perspective provides potential theoretical evidence for understanding the cellular interactions underlying acupuncture effects.

### 4.3 Secretion of chemical molecules and participation in acupuncture effects

A study conducted in 2010 reported that manual acupuncture can continuously induce the release of ATP (Goldman et al., 2010). The changes in adenosine concentrations can be quantitatively measured using micro dialysis. Targeted administration of adenosine A1 receptor agonists significantly inhibited pain perception in mice in the study. However, acupuncture effects were not observed in adenosine A1 receptor gene knockout mice (Goldman et al., 2010). This suggests that the adenosine A1 receptor may play a critical role in the peripheral mechanism underlying acupuncture-induced analgesia. The reorganization of the cytoskeleton induced in fibroblasts by acupuncture mechanical forces also involves purinergic signaling (Langevin, 2014). Adenosine signal transduction plays an analgesic role through neuromodulation (Langevin, 2014). ATP has Rho-dependent characteristics and can be released extracellularly during acupuncture. Based on this, adenosine release mediated by fibroblasts may propagate along the connective tissue plane, further explaining the distal effects of acupuncture and providing evidence for the direct involvement of fibroblasts in the mechanisms of action of acupuncture. In 2021, researchers confirmed that the TRPV4 mechanosensitive ion channel can induce the accumulation of extracellular ATP at acupuncture points in animal experiments, mediating the analgesic effects of acupuncture in an arthritis rat model (Zheng et al., 2021). The local analgesic effect of acupuncture seems to be more easily understood within the framework of biomechanics. For example, studies have shown that patients with chronic low back pain often have increased connective tissue thickness and poor connective tissue mobility, which can be objectively observed using assessments such as ultrasonography. The regulation of the ECM in fibroblasts by acupuncture can alter the mechanical environment of connective tissue and enhance its mobility, and exert an analgesic effect. This suggests that targeted stretching can induce fibroblast deformation and release adenosine, which reduces tissue pressure, increases shear motion, and ultimately alleviates pain. Studies have shown that electroacupuncture may enhance endogenous cannabinoids by upregulating CB2 receptors, activating the AMPK signaling pathway, and increasing the analgesic effects of β-endorphins in inflamed tissues (Lan et al., 2024). Current evidence suggests the presence of endogenous cannabinoid receptor 2 (CB2) in fibroblasts. However, the activation of CB2 receptors expressed by fibroblasts to exert analgesic effects via manual acupuncture requires further confirmation.

### 4.4 Change the physical environment, regulate pain perception

**FIGURE 4:**
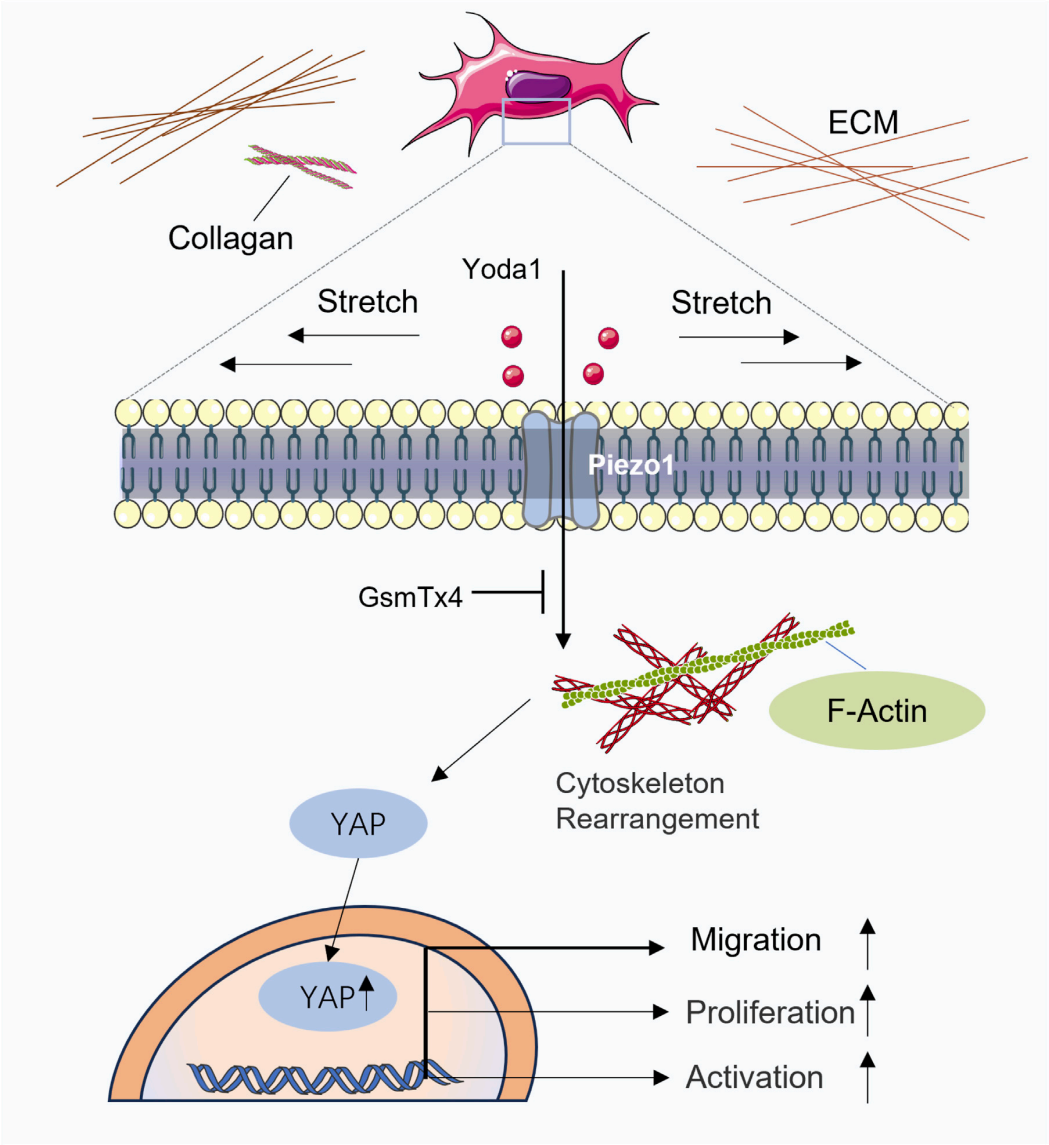
Schematic illustration of the mechanotransduction pathway. Reproduced from Tu et al. (2025), Biochim Biophys Acta Mol Cell Res, under CC BY license. Mechanical stimulation of fibroblasts induces cytoskeletal remodeling, leading to nuclear translocation of YAP and subsequent changes in fibroblast proliferation, migration, and differentiation. This mechanotransduction process may be mediated, at least in part, by activation of the Piezo1 mechanosensitive channels.

In traditional Chinese acupuncture theory, several Ashi points, which do not have fixed anatomical locations but are instead identified by their sensitivity to pressure, exist in addition to the acupoints located along the twelve meridians. Clinically, these points are often selected as primary targets for acupuncture-based analgesia. The concept of Ashi points closely parallels that of myofascial therapy trigger points, which are nodules located within the fascia between muscles. Pressure on these nodules can elicit referred pain, muscle spasms, and autonomic symptoms such as sweating and local temperature changes (Barbero et al., 2019; Mitidieri et al., 2020; Lee et al., 2022). Some scholars have explored the debate between dry needling and modern medicine from the perspectives of meridian theory and fascial theory, arguing that myofascial trigger points should not be equated with Ashi points in traditional Chinese medicine. The meridian theory and the myofascial framework have similarities, but they have significant conceptual and theoretical differences (Huang et al., 2018). Some studies have suggested that at least 93.3% of common myofascial trigger points correspond to traditional acupuncture points (Dorsher, 2009); however, further evidence is needed to support this association. Thomas Myers proposed that the fascial system of the body forms a continuous network that connects muscles and bones throughout the body. He described fascia as having a web-like appearance and capable of enveloping muscles, bones, nerves, arteries, veins, and lymphatic vessels (Myers, 2009). There appears to be a correlation between fascial structures and meridian pathways. Under the framework of modern biomechanics, interpreting the effects of acupuncture through fascial theory seems to be more convincing, and this is supported by the majority of researchers engaged in acupuncture studies both domestically and internationally (Langevin and Yandow, 2002; Wang et al., 2008; Jiang et al., 2009; Finando and Finando, 2011; Langevin, 2014; Langevin and Wayne, 2018; Guo Q et al., 2021; Zhou et al., 2022). It is indisputable that acupuncture induces measurable biological effects, whether interpreted through the lens of traditional Chinese meridian theory or modern fascial theory. While the relationship between myofascial trigger points and Ashi points will not be explored in depth in this article, we focus on identifying their shared features to elucidate the underlying biological mechanisms of acupuncture at such sites. Ashi points are typically characterized by pressure sensitivity and increased tissue tension, and experienced clinicians can often locate areas of injury through palpation alone. In traditional Chinese medicine, Qi is regarded as a vital energy maintaining bodily balance, and pain is often attributed to disruptions in the flow of Qi or blood. As described in the Huangdi Neijing: “Pain arises when there is blockage in the meridians or when the flow of qi within the meridians is insufficient (Bu tong ze tong; Bu rong ze tong).” This classical statement highlights the traditional Chinese medical perspective that pain results either from obstruction of the body’s channels or from inadequate nourishment and circulation within them. From the perspective of traditional Chinese medicine, tender points can be interpreted as pain responses caused by the stagnation of meridian Qi (Jing Qi). Acupuncture at these points, when accompanied by the sensation of De Qi, is believed to promote the flow of meridian Qi and relieve pain. In contrast, modern medical theory suggests that areas of tissue stiffness are primarily composed of ECM with excessive mechanical tension. This stiffness is largely attributed to the overaccumulation of ECM proteins mainly secreted by fibroblasts such as collagen, fibronectin, and hyaluronic acid. Therefore, deep fascia may serve as a potential anatomical substrate underlying the pain elicited by pressure at Ashi points (Weiss and Kalichman, 2021). Acupuncture induces the winding of collagen fibers around the needle, leading to mechanical coupling with extracellular matrix proteins such as fibronectin and laminin (Sun et al., 2025). This interaction alters the mechanical tension within the local microenvironment, which may partially account for the localized analgesic effects of acupuncture. For example, Fu’s subcutaneous needling (FSN) therapy represents an innovative approach grounded in the connective tissue theory. This technique specifically targets the loose subcutaneous connective tissue (Fu et al., 2007) with shallow needling. However, it can be effectively used to treat various conditions, including arthritis (Huang et al., 2024a), low back pain (Zhang et al., 2024), phantom limb pain (Huang et al., 2024b), cancer-related pain (Xu et al., 2023), and neck pain (Huang et al., 2022) through specific manipulations such as needle swaying. Fu’s subcutaneous needling operation has been shown to significantly alleviate sciatic nerve pain, reduce axonal swelling, and mitigate abnormal myelin and vacuolation of organelles. The mechanism underlying these effects is likely associated with the regulation of inflammatory pathways and endoplasmic reticulum stress pathways. A clinical report on the treatment of traumatic brain injury with floating needle therapy highlighted that the technique, which involved fascia sweeping and infusion methods applied to the fascia in the neck, orbital, and temporal regions, effectively improved diplopia and range of eye movement (Lu et al., 2024). The mechanism underlying this improvement is likely related to the release of fascial tension, reduction of nerve compression, and modulation of inflammatory factors. This suggests that floating needle therapy may have potential therapeutic effects for various conditions by relieving fascial tension and improving microcirculation. As shown in Figure 4 (Tu et al., 2025), simulated acupuncture mechanical forces induce changes in fibroblast cell area and cytoskeletal rearrangement, which in turn influence fibroblast proliferation, migration, and differentiation. While there is currently no direct evidence to confirm whether floating needle therapy induces microstructural changes in fibroblast area, cytoskeleton, or pseudopodia formation, it is reasonable to infer that any therapy targeting connective tissue could generate mechanical forces that alter the morphology of fibroblasts, given that fibroblasts are the most abundant cells in connective tissue.

Another piece of evidence that fibroblasts participate in altering the tissue microenvironment to influence pain perception is their ability to secrete components that make up the extracellular matrix. Hyaluronic acid is the most abundant polysaccharide in the extracellular matrix, and it facilitates tissue flexibility and acts as a lubricating substance to help maintain the movement of internal organs and deep fascia (Stecco et al., 2018). Hyaluronic acid is primarily synthesized and secreted by fibroblasts (Cowman et al., 2015). In addition to playing a role in regulating fascia structure, it is also associated with the development and progression of diseases such as cancer, diabetes, and inflammation (Liang et al., 2016; Marinho et al., 2021). Hyaluronic acid is heterogeneous; it has anti-inflammatory and immune-suppressive properties when its molecular weight is high, but it acts as a potent pro-inflammatory molecule when its molecular weight is low (Litwiniuk et al., 2016). Inflammatory and injury states are associated with increased concentrations of low-molecular-weight hyaluronic acid. Studies have shown that patients with cervical pain syndrome have increased thickness of connective tissue, restricted fascia mobility, and symptoms such as stiffness and muscle tension. These are primarily related to the elevated concentrations of low-molecular-weight hyaluronic acid, which reduce the lubricating properties of the fascia (Ugwoke et al., 2022). A study investigated the correlation between peripheral injury and fascia extracellular matrix remodeling using a unilateral sciatic nerve resection rat model. Peripheral nerve injury induced axonal rupture and it led to excessive collagen deposition and reduced hyaluronic acid concentration. These changes in the extracellular matrix are key contributors to tissue stiffness and functional impairment (Zhao C et al., 2024). Injury leads to extracellular matrix remodeling, and specific targeting of the extracellular matrix has potential therapeutic effects in pain intervention. Hyaluronic acid has been applied to the clinical treatment of pain and inflammatory diseases. For instance, injection of high-molecular-weight hyaluronic acid into the knee joint reduced the mechanical activation threshold of nerve endings in a gout and arthritis rat model. The underlying mechanism may involve the regulation of the TRPV1 mechanosensitive ion channel (Marcotti et al., 2018). Another independent study explained that high-molecular-weight hyaluronic acid can directly act on the TRPV1 channel, stabilizing its closed conformation. This reduces the response to thermal and capsaicin stimuli and decreases the firing frequency of action potentials in neurons of the dorsal root ganglion. As a result, it exerts an analgesic effect in the arthritis mouse model (Caires et al., 2015). It is evident from the studies mentioned that fibroblasts secrete extracellular matrix components such as hyaluronic acid, fibronectin, and collagen under mechanical force stimulation; these contribute to the formation of the extracellular matrix. Changes in the extracellular matrix result in alterations of the physical properties of the tissue microenvironment, and these affect pain perception. This suggests that physical interventions targeting fibroblasts or the fascial system constructed by fibroblasts can be effective. This helps explain the biological mechanisms underlying the effects of physical therapies such as ultrasound, massage, and acupuncture in reducing tissue tension and pain. It also highlights the potential of research into physical interventions targeting fibroblasts for tissue repair, regeneration, and medical aesthetics.

### 4.5 Construct a tension system and participate in whole body regulation

As stated in the Biaoyou Fu: “In cases of intersecting channels, one may needle contralaterally—when there is illness on the left, treatment is applied on the right; in draining the collaterals, distal needling is employed—when there is illness in the head, treatment is applied to the foot.” This classical description reflects the traditional acupuncture principle that symptoms are not always treated locally, but may be alleviated by stimulating distal or contralateral points along interconnected meridians. This refers to the method of Miao needling recorded in the Huangdi Neijing, where diseases on one side are treated by needling the opposite side to regulate the balance of Qi and blood in the meridians. The Miao needling method is widely applied in clinical practice and is known for its therapeutic outcomes (Lei et al., 2018; Hao, 2023). For example, lateral epicondylitis can be treated by needling the wrist and upper limb acupuncture points (Shin et al., 2013), phantom limb pain can be alleviated by needling the healthy side of the body (Guo Q et al., 2021), and headaches can be treated by needling points on the hand (Kaptchuk, 2002). Those with a background in traditional Chinese medicine may explain these phenomena using meridian circulation and Qi conduction theories. However, this phenomenon needs to be explained within the framework of modern scientific systems and beyond the concept of meridians for modern physiologists and pathologists who emphasize logic and objective evidence. Some researchers have used MRI to clarify the effects of distal stimulation on the brain and found that the biological mechanism of the Miao needling theory may be related to neural regulation and brain network function modulation (Li et al., 2019). Modern molecular biology and biomechanics may provide partial references to address this issue (Ingber, 2018). As mentioned earlier, fibroblasts have gap junctions that allow them to couple and form a cellular network. This feature facilitates the response of fibroblasts to mechanical signals and enables spontaneous calcium oscillations and synchronized contractions to be transmitted across the network (Camelliti et al., 2004; Langevin et al., 2004; Follonier et al., 2008; Li et al., 2011; Lembong et al., 2015; Murata et al., 2019). Fascia theory researchers believe that the human fascia is a unified system that plays a key role in maintaining structural stability, transmitting forces, and supporting the structure and movement of organs and tissues (Zügel et al., 2018). The tension system of the fascia is primarily composed of fibroblasts (Tomasek et al., 2002). Factors such as injury, compression (Sasabe et al., 2017), and even obesity (Wan et al., 2021) can alter the mechanical environment of fibroblasts, leading to their transformation into myofibroblasts, which can cause tissue contraction or increased tension. Modifying the tension of the fascia can serve as a target for pain and neural regulation (Wu et al., 2021; Du et al., 2023; Gordon et al., 2024), with applications in the management of various diseases such as pain and muscle tightness (Plaut, 2022). For example, fascia therapy supports the regulation of distal tension structures to improve the range of motion or pain perception at the affected area in the case of pain. Research has shown that heel pain is related to lower back pain and lumbar dysfunction (McClinton et al., 2018), and treating the lower back may effectively alleviate plantar pain. Similarly, acute stretching of the lower limbs can influence the range of motion in the upper limbs and *vice versa* (Nordez et al., 2017). The fascia is an integrated network composed of fibroblasts. Acupuncture can regulate the tension structure of the fascia and exert systemic or distal therapeutic effects (Plaut, 2023). In the case of connective tissue disorders such as fibromyalgia, acupuncture at different body locations can adjust the bio-tensile system of fascia and reduce its shear modulus. This, in turn, influences the fibroblasts in the fascia through mechanical sensitivity signals and changes in stiffness (Sánchez-Infante et al., 2021).

Another key mediator of remote signaling is extracellular vesicles, which have diameters of 30–200 nm (Li et al., 2022). All cell types can secrete extracellular vesicles (Bobrie et al., 2011). As important carriers of cellular communication, they can transmit proteins and genetic information to target organs and play a crucial role in antigen presentation, immune responses, and other processes (Whiteside, 2016). In our previous studies, we found that the serum exosomes produced after acupuncture can effectively improve lung injury in septic mice and downregulate pro-inflammatory cytokines in the serum (Zhang et al., 2023). The mechanism may be related to the involvement of exosomes in macrophage polarization and their targeting of disease sites. The inhibition of exosomes can reduce the anti-inflammatory effects of acupuncture (Zhuo et al., 2023). Another study reported that the NLRP3 inflammasome can activate the release of inflammatory exosomes into lung tissue, and electroacupuncture can improve lung injury by inhibiting the activation of the NLRP3 inflammasome (Zou et al., 2021). These three studies confirm that acupuncture can participate in the regulation of exosomes and exert therapeutic effects on various diseases. Exosomes from fibroblasts contain various bioactive substances, including extracellular matrix components (such as collagen, fibronectin, and hyaluronic acid), nucleic acids, and lipids. These molecules facilitate signaling between fibroblasts and other cells, regulating the functions and behaviors of target cells. Exosomes derived from fibroblasts have potential roles in repairing skin damage caused by photoaging (Hu et al., 2019). Tumor-associated fibroblast-derived exosomes may also influence tumor cell proliferation, invasion, and metastasis (Shen et al., 2021). The research conducted by the team of Carla Stecco involved the use of a CB2 receptor agonist to intervene in human fascial fibroblasts. The Study has shown that CB2 receptors are expressed in fascial fibroblasts, and their activation is associated with the production of hyaluronan-rich vesicles, which may play a crucial role in the lubrication and functional integrity of fascial tissues. Furthermore, activation of CB2 receptors may modulate the activity of fascial fibroblasts, thereby contributing to the processes of tissue repair and regeneration (Fede et al., 2020). They found that vesicles enriched with hyaluronic acid were produced in the extracellular space and rapidly released into the extracellular environment after 4 h of drug treatment. Furthermore, the blockade of CB2 receptors inhibited the production of extracellular vesicles. This study revealed that fibroblasts can secrete vesicles rich in extracellular matrix molecules, which play a role in cellular communication and tissue repair and regeneration. Direct evidence linking the effects of acupuncture to the regulation of fibroblast-derived extracellular vesicles is lacking. However, existing evidence on the promotion of the release of extracellular vesicles by acupuncture and the roles of fibroblast-derived vesicles in tumor metastasis and tissue repair provides a theoretical framework for further research into the involvement of fibroblast extracellular vesicles in the mechanisms of action of acupuncture.

## 5 Discussion

This study systematically integrates evidence on cell biology, biomechanics, and molecular signaling pathways and reveals the potential role of fibroblasts as key intermediaries for the effects of acupuncture. The mechanosensitive properties of fibroblasts make them crucial mediators in the conversion of mechanical signals from acupuncture to biochemical signals. This provides a biologically grounded explanatory framework for acupuncture therapy. The shear forces generated by acupuncture activate the Rho/ROCK pathway through the integrin-cytoskeleton system, aligning with the clinical phenomenon of “deqi,” where the needle is passively pulled during acupuncture, and local tension changes occur (Briggs and Shurtleff, 2017; Song et al., 2023). It also helps explain dry needling therapy, where local muscle contractions are leveraged as indicators of acupuncture effectiveness (Liu et al., 2014; Guo Q et al., 2021). The local tension changes and sensory nerve activation accompanying the “deqi” sensation are key elements of the effects of acupuncture (Lu et al., 2021; Li B et al., 2023; Zhong et al., 2024). At the same time, the YAP/TAZ signaling axis is a core pathway for cells to respond to mechanical signaling and transduction (Zhong et al., 2023; Mei et al., 2024; Zhao X et al., 2024). Its mechanosensitive activation provides the molecular basis for the efficacy of acupuncture in connective tissue disorders. For instance, the activation of this pathway can alter tissue Young’s modulus and influence the mobility of the damaged area. This mechanism underscores how acupuncture may influence tissue properties at the cellular level and aid in the restoration of normal tissue function by modulating the mechanical properties of the extracellular matrix and surrounding tissues. In clinical practice, the evaluation of connective tissue and fascia largely relies on manual palpation by clinicians, which is not objective and is based on quantifiable biological markers. High-precision technologies such as nanoindentation and atomic force microscopy can measure ECM stiffness, but their direct application in clinical settings is challenging. Technologies like shear wave elastography, magnetic resonance imaging, and pressure gauges can quantitatively measure tissue elasticity modulus and shear modulus *in vivo* and are promising for precision acupuncture therapy based on fibroblasts. These technologies also open new avenues for objective diagnosis and efficacy validation of meridians and acupoints (Chao et al., 2018; Götschi et al., 2023). This integration of advanced imaging and mechanical measurements may enhance the precision of acupuncture treatments and facilitate the objective evaluation of therapeutic effects.

**FIGURE 5:**
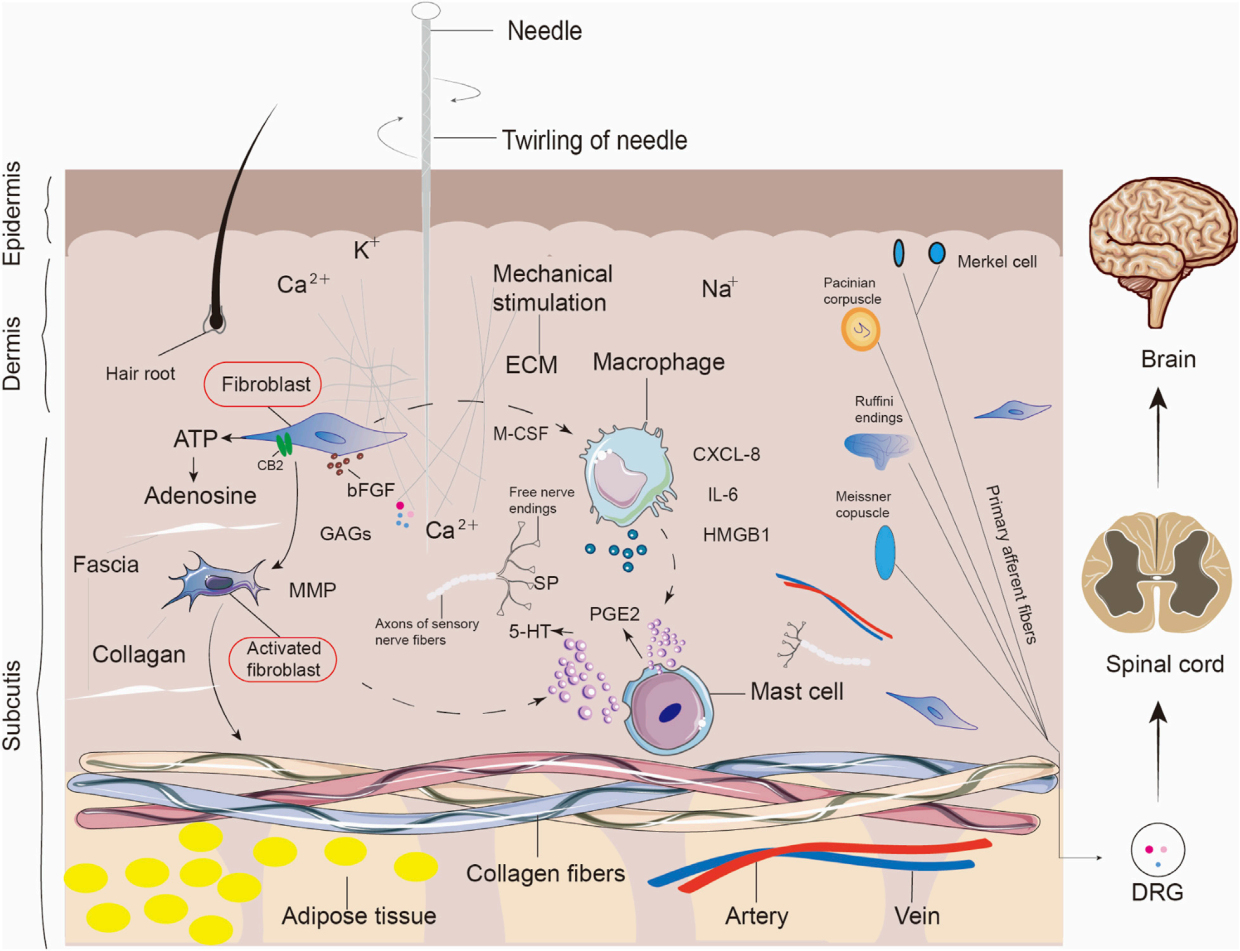
Cellular and molecular responses to acupuncture-induced mechanical stimulation at acupoint sites. This schematic illustrates the complex microenvironmental changes that occur in the skin, fascia, and underlying tissues following acupuncture needle insertion and twirling. Mechanical stimulation activates various local cell types, including fibroblasts, macrophages, mast cells, and nerve endings. In the fascia, fibroblasts respond to ECM deformation and release MMPs, bFGF, and GAGs, contributing to tissue remodeling. Macrophages are activated and secrete pro-inflammatory mediators such as IL-6, CXCL-8, and HMGB1. Mast cells degranulate and release PGE2, serotonin (5-HT), and SP, facilitating neuroimmune crosstalk. ATP and adenosine are released locally and may act through CB2 and purinergic receptors. Sensory structures including Merkel cells, Pacinian corpuscles, Meissner corpuscles, and Ruffini endings transduce mechanical signals to the spinal cord and brain via primary afferent neurons and DRG. These events collectively contribute to initiating neuromodulatory and immunoregulatory responses triggered by acupuncture.

Fibroblasts may not only achieve “non-neuronal analgesia” through the secretion of adenosine and regulation of endogenous cannabinoid receptors or hyaluronic acid (Goldman et al., 2013; Langevin et al., 2013; Lembong et al., 2015; Fede et al., 2020; Wu et al., 2024), which provides a molecular basis for the traditional acupuncture theory of “regulating Qi.” They may also release other neurotransmitters to mediate analgesic and anti-inflammatory effects. These potential mechanisms need to be validated through advanced techniques like single-cell sequencing and genomics. Such studies would deepen our understanding of how fibroblasts contribute to the therapeutic effects of acupuncture beyond the conventional concepts of Qi regulation and shed light on novel molecular pathways that can enhance the efficacy of acupuncture in pain management and inflammation control. Single-cell interaction analysis reveals the recruitment of macrophages by fibroblasts through chemokine pathways and the dual role in immune regulation and tissue repair. This interaction is crucial in maintaining tissue homeostasis and promoting healing. By secreting specific chemokines, fibroblasts can guide the migration of macrophages to sites of injury or inflammation, where they collaborate in resolving inflammation and initiating tissue regeneration. These findings underscore the importance of fibroblasts not only as structural cells in connective tissue but also as active participants in immune responses and tissue repair processes (Alexanian et al., 2024; Zhou et al., 2024). A lot of tumor-related research has revealed the interaction patterns between fibroblasts and macrophages, and their specific roles in acupuncture efficacy are equally deserving of further exploration. As shown in Figure 5, fibroblasts may be the first responders to mechanical stimuli after acupuncture, and they trigger a cascade of multi-step reactions that include macrophage polarization, mast cell degranulation, and activation of mechanosensitive ion channels (Yao et al., 2014; 2024; Li S et al., 2023). In the context of these existing theories, the present study highlights the role of fibroblasts as crucial effectors in acupuncture-induced responses. Unlike neural or humoral pathways that act primarily through systemic modulation, fibroblasts provide a structural and cellular basis for local mechanotransduction. When acupuncture needles are manipulated, fibroblasts in the surrounding connective tissue sense and respond to mechanical forces, leading to cytoskeletal remodeling (Langevin et al., 2006; Yunshan L et al., 2025), extracellular matrix reorganization (Tu et al., 2025), and the secretion of bioactive molecules such as cytokines, growth factors, and hyaluronic acid. These responses can modulate immune cell activity, promote tissue repair, and alter local nociceptive signaling. Therefore, fibroblasts may represent a central cellular mechanism that bridges mechanical stimulation at acupoints with downstream effects relevant to pain, inflammation, and tissue homeostasis. By positioning fibroblasts within the broader theoretical framework of acupuncture mechanisms, this study provides novel insight into their potential roles across multiple disease contexts, including inflammatory pain, fibrotic disorders, and wound healing. However, the relative contribution of each cell type to the overall effect remains inconclusive. To this end, we advocate for the establishment of an acupoint cell atlas to systematically quantify the involvement of different cell types in the acupuncture effect. This approach would facilitate the precise analysis of the mechanisms of acupuncture and promote its clinical applications. By mapping the cell types and their interactions at acupoint regions, we can better understand how acupuncture induces therapeutic effects at the cellular and molecular levels and pave the way for more targeted and evidence-based acupuncture treatments.

Fibroblast-derived exosomes may carry mechanistic information related to mechanical stimuli and participate in the distal regulation advocated for the “Miao Acupuncture” method. However, this biological pathway remains to be experimentally validated. The role of fibroblasts in the mechanism of action of acupuncture relies on the continued advancement of translational medicine. For example, high-molecular-weight hyaluronic acid has been widely used in the treatment of arthritis. Several clinical studies have shown that hyaluronic acid alone or in combination with acupuncture can significantly alleviate joint pain and inflammatory responses (Caires et al., 2015; Xiao et al., 2019; Bonet et al., 2023). The application of recombinant type IV collagen in medical aesthetics has also confirmed its value in tissue repair and remodeling (Salvatore et al., 2023). These findings suggest that targeting fibroblasts with biologics or small-molecule drugs can further enhance the therapeutic effects of acupuncture. Moving forward, new fibroblast regulators can be developed to strengthen and expand the clinical applications of acupuncture therapy. This represents a promising avenue for improving tissue healing and regeneration, as well as addressing conditions that involve fibrotic remodeling or chronic inflammation.

The network formed by fibroblasts through gap junctions in the fascia can transmit long-range signals; however, its spatial topology has not yet demonstrated the same specificity as meridian pathways. On the other hand, fibroblasts in acupoint and non-acupoint areas may differ in heterogeneity, spatial distribution, and their interactions with blood vessels and nerves, but the supporting evidence remains insufficient. Future research should integrate spatial transcriptomics and biomechanical modeling and utilize artificial intelligence to analyze data such as cell morphology, gene expression, tensile forces, and tissue stiffness (Oria et al., 2025). This approach can help predict fibroblast responses to mechanical stimuli. It can also provide insights into key aspects such as the heterogeneity of fibroblasts in acupoint areas and the pathways of acupuncture mechanical signal transmission to improve our understanding of acupuncture.

## 6 Conclusion

This study demonstrates that fibroblasts contribute to the effect of acupuncture through a three-tiered response mechanism involving mechanical perception, signal transduction, and system regulation. It systematically elucidates the central role of fibroblasts in the mechanism of action of acupuncture. The findings will facilitate a modern biological interpretation of traditional acupuncture theory and provide a theoretical foundation for the development of novel drugs or physical therapies targeting fibroblasts.
